# Examining Cognitive Factors for Alzheimer’s Disease Progression Using Computational Intelligence †

**DOI:** 10.3390/healthcare10102045

**Published:** 2022-10-17

**Authors:** Fadi Thabtah, Swan Ong, David Peebles

**Affiliations:** 1 ASDTests, Auckland 0610, New Zealand; 2 Digital Technologies, Manukau Institute of Technology, Auckland 0481, New Zealand; 3 Department of Psychology, University of Huddersfield, Huddersfield HD1 3DH, UK

**Keywords:** Alzheimer’s Disease (AD), clinical informatics, cognitive informatics, classification, dementia, machine learning, neuropsychological assessments

## Abstract

Prognosis of Alzheimer’s disease (AD) progression has been recognized as a challenging problem due to the massive numbers of cognitive, and pathological features recorded for patients and controls. While there have been many studies investigated the diagnosis of dementia using pathological characteristics, predicting the advancement of the disease using cognitive elements has not been heavily studied particularly using technologies like artificial intelligence and machine learning. This research aims at evaluating items of the Alzheimer’s Disease Assessment Scale-Cognitive 13 (ADAS-Cog-13) test to determine key cognitive items that influence the progression of AD. A methodology that consists of machine learning and feature selection (FS) techniques was designed, implemented, and then tested against real data observations (cases and controls) of the Alzheimer’s Disease Neuroimaging Initiative (ADNI) repository with a narrow scope on cognitive items of the ADAS-Cog-13 test. Results obtained by ten-fold cross validation and using dissimilar classification and FS techniques revealed that the decision tree algorithm produced classification models with the best performing results from the cognitive items. For ADAS-Cog-13 test, memory and learning features including word recall, delayed word recall and word recognition were the key items pinpointing to AD advancement. When these three cognitive items are processed excluding demographics by C4.5 algorithm the models derived showed 82.90% accuracy, 87.60% sensitivity and 78.20% specificity.

## 1. Introduction and Background

There are 50 million people worldwide living with dementia, a disease that has a devastating impact on a person’s physical and psychological status, as well as damaging effects on society and the economy [1]. Dementia is a neurodegenerative disorder characterised by symptoms and signs exhibiting difficulties in memory, disturbance in language, psychological and psychiatric changes, and impairments in activities of daily living, mainly occurring in the elderly [2]. In the United Kingdom, there is an estimate of 885,000 people living with dementia in 2019, and it is projected to increase by 80% to around 1.6 million people in 2040 [3]. To date, there is yet a cure or an effective treatment for the disease. Particularly in the times of a pandemic where focus and resources are being diverted, it is only crucial that a fast, affordable, and reliable way for diagnosis and prognosis using innovative technologies such as machine learning and artificial intelligence, is devised.

Identifying few cognitive and functional features that causes progression of the disease can assist in early intervention [4,5]. While pathological assessments of AD diagnosis such as biological markers (biomarkers), cerebrospinal fluid (CSF), magnetic resonance imaging (MRI), can be used to predict the disease, they are also time and cost intensive, stressful, results requiring laboratory study and professional personnel which may not be available most of the times [6,7]. Neuropsychological assessments such as the FAQ, the ADAS-Cog-13, Mini Mental State Examination (MMSE) and others [8,9,10], are useful methods that can screen for signs of early cognitive impairment. These assessments are usually quicker, easier to carry out when compared with pathological procedures and show good performance with reference to validity, sensitivity, and specificity [11,12]. However, little research works have been conducted on measuring the progression of AD using cognitive features, i.e., [6,7,13]. Below some of the relevant research works are reviewed and analysed to pinpoint to the gaps that this research dealt with.

Wessels et al. [4] aimed to identify a composite scale called the Integrated Alzheimer’s Disease Rating Scale (iADRS), that can measure the impactful domains of AD, which combines cognition and function through the evaluation of existing scales. The datasets utilised to assess the iADRS were from the longitudinal studies of ADNI [14], and clinical trials of anti-dementia drugs such as Solanezumab, Semagacestat and Donepezil. Due to the various scales difference in total points in relative to decline, signal-to-noise ratios (SNRs) were calculated for comparability. Principal component analysis (PCA) was used to establish the psychometric properties of the iADRS, assessing the contributions of the two scales total scores, and the contribution of individual item scores. Their results found that composites combining cognition and instrumental Activities of Daily Living items are better at detecting the disease than traditional cognitive-only or functional-only scales across MCI, mild AD, and moderate AD. As for item analyses, they discovered that delayed recall, word recall and word recognition items were the most sensitive cognitively, and reading item was the most sensitive functionally.

Podhorna et al. [15] compared the performance of the 3-, 5-, 11- and 13-item ADAS-Cog variant subscales using ADNI data, that can best detect cognitive decline. The original 11-item version of ADAS-Cog was to measure cognition in patients with mild to moderate AD, but lack the capability to detect change and measure cognitive domains known to cause impairment in early stages of AD. Thus, the creation of new ADAS-Cog variants such as the 13-item subscale to improve its properties for early AD screening with the use of additional tests such as digit cancellation and delayed word recall. As a baseline comparison, the authors have assumed the ADAS-Cog 11 score of 10 (out of 70) for subjects with MCI, and a score of 18 (out of 70) for subjects with mild AD. Based on their findings, the ADAS-Cog13 score of 15 (out of 85) was considered MCI, while a score of 30 (out of 85) was considered mild AD. Overall, they concluded that the impact of expansion or reduction of the ADAS-Cog was subtle, but noted that in mild AD, adding items appeared to provide more benefit than removing items.

Battista et al. [7] conducted a machine learning study to assess cognitive, behavioural, and functional measures and its potential in reducing the amount of neuropsychological test used to improve the classification of AD patients, and at an early stage of impairment. They aimed to explore using more subdomains that is concerned with long-term memory and recognition memory. While the study had also mapped the features to DSM-5 cognitive domains [16], each feature was only limited to one domain when certain tasks could involve more than one. It would have been helpful to understand the method they have used to assign the domains. The DSM-5 framework introduced 6 cognitive domains: complex attention, learning and memory, perceptual-motor control, executive function, language, and social cognition. The study utilised neuropsychological tests and features obtained from the ADNI database. Two feature reduction approaches were used to improve computational performance: (a) a computational approach, and (b) a clinician’s understanding based on their expertise and experience. With the reduced set of features, a classification algorithm based on Support Vector Machines (SVMs) was used to train the dataset into predicting the likelihood of being diagnosed with AD. Overall, using the computational feature reduction approach, items that are relevant to our research that were found to be the best predictors were ADAS Q1, Q4, Q8, FAQFORM and FAQREM. Similarly, using the clinician’s reduction approach, ADAS Q1, Q4, Q8 was found to be the best predictor, and due to the FAQ items having overlapping measures, they have been excluded.

Pereira et al. [11] explored the different FS techniques in predicting the advancement of the AD disease. The study aimed to derive subsets of vastly reduced features from neuropsychological tests, using a FS ensemble technique that combines both stability and predictability. Ref. [11] combined seven methods based on different strategies to measure the impactful features, and then paired with different classifiers to observe which combination attained the best classification performance. They found that Naïve Bayes algorithm was the strongest performer and Decision Tree the weakest in terms of predictive accuracy. The training dataset was retrieved from ADNI and the Portuguese Cognitive Complaints Cohort, both achieving relatively good model performance at AUC above 0.87 and 0.82 respectively. For the ADNI dataset, the top selected features for ADAS were ADAS-Cog Total 13, ADAS-Cog Total 11, ADA-cog Q4, ADAS-Cog Q8 and ADAS-Cog Q1. The results were comparable to [7], where the study had also identified Q4, Q1 and Q8 to be the top features in identifying disease progression.

Albright [17] set out to predict the progression of AD using a neural network model based on the ADNI dataset, using a longitudinal analysis methodology where the data was pre-processed for missing values or misaligned data type, and divided into 3 sets (LB1, LB2 and LB4). LB1, used as a training and validation dataset and was processed using the all-pairs technique, and then classified using various machine-learning classifiers. Each classifier was evaluated using the 7-fold cross-validation. A receiver operating characteristic area under the curve (ROC-AUC) was also used to measure the effectiveness of each classifier. Ref. [17] found that multilayer perceptron neural networks and recurrent neural networks had the best performance in cross-validation studies. Ref. [17]’s model was 86.6% effective at predicting the progression of AD, either from a state of clinically normal (CN) or MCI. While this study illustrated the success of predicting disease progression, it did not explain specifically on how the three set of features training group were derived.

Kueper et al. [18] brought to light that there are as many as thirty-one modified versions of ADAS-Cog available to assess the level of cognitive dysfunction in Alzheimer’s disease. The authors set out to review the performance of the different versions of ADAS-Cog at detecting the progression of dementia in both dementia and pre-dementia populations. A limitation in that was that the large variety of modified versions is an issue for cross-study comparisons. This was useful to this research in that it brought to our attention the various versions available, so their differences were studied. For our research, the interest is in the findings of ADAS-Cog-13 [19] as our dataset retrieved from ADNI utilises this version. Ref. [19] deduced that the ADAS-Cog-11 did not assess attention and concentration, planning and executive function, verbal memory, nonverbal memory, and praxis. Therefore, Ref. [19] recommended to include, in addition to the ADAS-Cog-11 items, a test of delayed word recall and number cancellation, which became the ADAS-Cog13, and with an altered scoring range of 0 to 85. Ref. [18] derived that the ADAS-Cog-13 ability to identify disease progression was better than that of ADAS-Cog-11.

Thabtah et al. [20] investigated the mapping between elements of common cognitive dementia diagnosis methods and the DSM-5 framework trying to identify methods that cover more cognitive domains, after thoroughly investigating digital medical questionnaires related to dementia screening in a previous research study, i.e., [21]. Recently, Thabtah et al. [5] examined experimentally the items of the Functional Activities Questionnaire (FAQ) using real data from ADNI-FAQ data sheet aiming to identify dissimilar functional elements that may deteriorate early during the progression of the AD. The authors utilised FS methods with classification algorithms to achieve the aim after balancing the dataset. The results revealed few sets of functional items including Administration and Shopping that have good correlations with the diagnostic class label. Predictive models constructed by the classification algorithms from the distinct items’ subsets of the FAQ sheet showed acceptable accuracy, sensitivity, and specificity rates.

The above studies acknowledged that the early detection and distinguishing the stages of AD were important for appropriate treatment purposes but did not consider the prospect of each cognitive items to identify disease progression to assist early intervention. While Battista et al. [7] used machine learning to assess cognitive, behavioural, and functional measures, it only focused on diagnosing AD and not the progression of the disease, which is more challenging. Furthermore, a mapping between the assessed items and the Diagnostic and Statistical Manual of Mental Disorders (DSM-5) framework [16], would have been helpful for the clinician’s understanding of linking the machine learning results and actual diagnosis of the disease besides the key areas that might pinpoint to the disease development [20]. Therefore, to fill the gap, this research investigates the problem of identifying key cognitive items that can primarily trigger the advancement of the AD using a data driven approach. The focus is on a widely accepted neuropsychological test that emphasise on cognition activities (ADAS-Cog-13 [9]).

To deal with the problem, the aim is to propose a data driven methodology that incorporates machine learning technology using real datasets from ADNI [14] (ADNIMERGE, ADAS-Cog13). Since the problem is to detect the progression of the AD using specific features, this can be seen as a supervised learning problem in data science in which a classification technique is utilised to construct classification models. These models in turn will be exploited by the clinicians or diagnosticians to detect any possible advancement of the AD using only few yet effective items. The scope of our research is limited to cognitive assessments in ADNI repository of ADAS-Cog13, therefore results related to other neuropsychological tests or biomarkers are excluded. The research questions that this research study is trying to answer are:

- How can we find cognitive items of ADAS-Cog13 that may be used as indicators for the possibility of AD advancement using classification models with feature selection techniques?

This study contributes the following benefits to clinicians:

1. The ability to predict progression of AD using models derived from small sets of features by using computational intelligence methods. These models can be incorporated into digital platforms for accessibility, fast and accurate screening in busy clinics for quick referrals.
2. Present few impactful cognitive items that are crucial to disease progression
3. The association of the cognitive items to the DSM-5 framework can be provided as a digital information sheet to the clinicians as a knowledge base toolkit during early screening.

Recent works on functional assessment using data driven approach to detect dementia advancement, e.g., [5], have been extended by incorporating new cognitive items from the ADAS-Cog-13. Hence, new impactful cognitive items can be detected early to indicate dementia progression if any. These are crucial to disease progression where clinicians can use this knowledge in early screening and can help in improving the design of future neuropsychological assessments.

The structure of this research paper is as follows: Section 1 introduced the background of the study along with review of related works to dementia research that utilised machine learning techniques, assessing cognitive items, using the ADNI data repository. Section 2 presents a research methodology. Section 3 describes experimental settings, and Section 4 displays the results. The discussion is presented in Section 5, and lastly conclusions are given in Section 6.

## 2. Methodology

**Figure 1:**
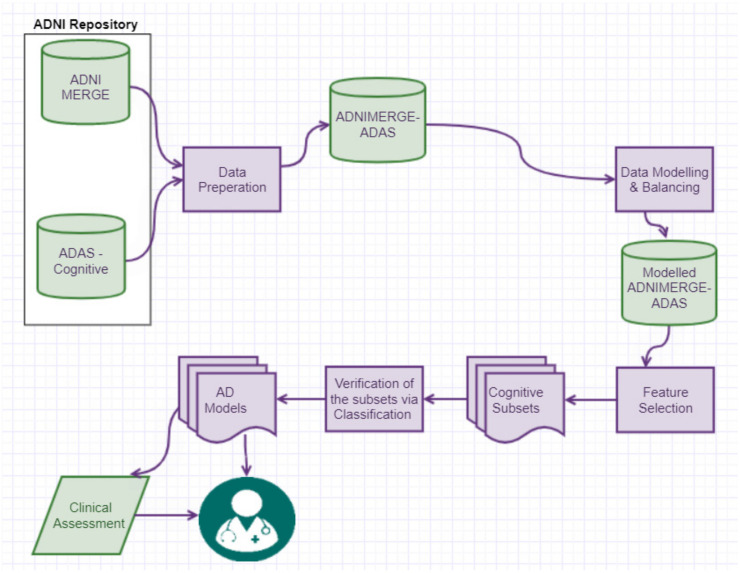
Methodology followed.

Figure 1 illustrates the methodology followed to answer the research question, which is “How the key cognitive items of ADAS-Cog13 that trigger the progression of AD can be found using computational intelligence techniques?”. Literature review in Section 1 was conducted to facilitate domain and data understanding, as well as familiarising with the metadata [14] were vital to assist our data preparation process. The approaches involved were data retrieval, integration, pre-processing, modelling, data balancing, data analysis using machine learning, and finally the best performing classification model is selected to form screening sheets which a clinician can use to predict the likelihood of a patient’s cognitive-function declining. A similar data driven methodology such as the one of [5] was followed.

### 2.1. Medical Test Used

The ADAS-Cog13 comprises of cognitive tasks that assess few cognitive domains like memory, communication and orientation among others [9]. There are as many as 31 variants to the ADAS test [18], the version analysed in this research study is the ADAS-Cog13 [19], which is utilised in the ADNI study where data were retrieved for processing. The ADAS-Cog13 consists of 13 items with a few tasks having a slightly different scoring range. The total ADAS-Cog13 scores range from 0–85, with the largest score indicating significant impairment. Ref. [15] suggested that a score of 15 was considered mild cognitive impairment (MCI), while a score of 30 was considered mild AD. As Ref. [18] acknowledged a limitation to the ADAS-Cog assessment is the various versions available that makes it difficult for cross-comparison of its validity and reliability. The most frequently used assessment in validation studies is the ADAS-Cog, where for MCI patients, the accuracy is of 82–83%, sensitivity 58–61% and specificity of 91–93%; and for AD patients, the accuracy is of 90.5–99.6%, sensitivity of 74–94% and specificity of 92–98% [22,23].

Multiple studies have consistently referenced their findings to cognitive domains in particularly executive function, memory, and attention to provide the association to the DSM-5 framework, i.e., [4,7,15,24].

### 2.2. Data Used and Integration Process

The datasets used in our methodology were retrieved from the ADNI database repository (http://adni.loni.usc.edu). The ADNI project started in 2003 and was led by Michael W. Weiner, MD. The main goal of the project was to evaluate whether MRI, PET, biomarkers, and other neuropsychological tests can be integrated to quantify MCI or AD progression. Please check www.adni-info.org for more information.

ADNI is a longitudinal multi-site study that collects, validates, and utilises data to develop clinical, imagine, genetic, and biochemical biomarkers for the early detection and tracking of AD [14]. It has conducted four studies: ADNI-1, ADNI-Go, ADNI-2 and ADNI-3, each having different goals, duration, funding, and group of participants. The study has a cohort of 1900 participants, mainly based in USA and Canada. The study is longitudinal as each participant are being monitored on a six-monthly basis to track and study AD progression, and thus have multiple observations per patient but at different points in time. Table 1 presents the description of the retrieved datasets before pre-processing. Two datasets, ADNIMERGE, and ADAS-Cog were retrieved from the ADNI database repository.

The ADNI-Merge dataset is an amalgamation of data from the four studies: ADNI-1, ADNI-Go, ADNI-2 and ADNI-3. The merged dataset has 14,627 data examples and 113 items related to patients’ visits, cognitive tests, functional questionnaires, genetic, demographics, biomarkers, and the diagnostic class (DX) among others. In this dataset, only 9 attributes are relevant to our experiment, they are the patient ID (RID), time of visit (VISCODE), diagnosis (DX) and demographics (AGE, PTGEN, PTEDUCAT, PTRACCAT, and PTMARRY).

ADAS-Cog dataset details the patient’s score in each of the 13 tasks and the total scores attained during the assessment. It contained 6770 observations and 121 attributes. In this dataset, only 15 attributes are relevant to our experiment, they are the patient ID (RID), time of visit (VISCODE2), and the scores of the questions (items), i.e., Q1SCORE, Q2SCORE, Q3SCORE, Q4SCORE, Q5SCORE, Q6SCORE, Q7SCORE, Q8SCORE, Q9SCORE, Q10SCORE, Q11SCORE, Q12SCORE and Q13SCORE.

Irrelevant features that are not the focus of our research such as patient information and biomarkers were filtered out from each dataset. All missing values such as incomplete individual task scores or missing diagnosis (DX) values, were dealt with mainly by way of removal. It was important to cleanse the datasets individually before integration to maintain decent computational performance.

Data integration is performed to produce a central dataset that will addresses cognitive elements (ADNIMERGE-ADAS). For the integration, the RID: patient ID and VISCODE: visit code were used as a reference for the merger. The purpose of the merging is to collect individual cognitive elements’ scores from the respective dataset, and the diagnostic class (DX) from ADNIMERGE for each visit per patient. There were data instances where a patient observation in the ADNIMERGE was not integrated due to the ADAS-Cog dataset not having a corresponding RID and visit code. This could be due to the assessments not being performed for the patient during a visit for various reasons. Thus, no merging occurs, resulting in lesser observations in the new dataset.

### 2.3. Diagnostic Class Data Modelling and Data Balancing

As the key element to our research is measuring the disease advancement, the DX attribute originating from the ADNIMERGE dataset is the key to our data modelling process since it represents the current diagnosis of the individual. A similar process of [5] for data modelling is followed in which two new attributes are created to establish a progression class attribute for each patient and their subsequent visits. The following steps to generate the DX Progress target class is performed:

1. The merged dataset is sorted by RID and then VISCODE2.
2. The first created is called ‘DX Digit’ which will map the DX attribute using a numeric label whereby CN = 1, MCI = 2, 3 = AD.
3. The second new attribute to be created is called ‘DX Progress’ which its purpose is to detect when the diagnosis is changed for an individual, establishing the target attribute. The DX Progress attribute will model the changes of the DX digit from the matching patient ID and their subsequent visit with three possible class values. Any progression from CN to MCI or MCI to AD will be labelled 1 in the ‘DX Progress’. When there is no disease advancement, the attribute is labelled as 0; any possible regression is labelled as −1.
4. For each patient’s baseline visit, the DX progress will be labelled as ‘0′ to ensure that the class does not model a previous observation that does not have a matching patient ID (RID).

Once the new class (‘DX Progress’) is formed, instances that has been assigned with regression ‘−1’ were removed, as the focus is on class labels that are either ‘1’ for progression or ‘0’ for no progression, with only two class values remaining. Table 2 shows features and data statistics.

The class set ‘DX Progress’ in the dataset after integration and modelling is imbalanced due to the significantly high number of no progression (0) class versus progression (1). Proceeding with an imbalanced dataset will produce a bias analysis that prefers the largest frequency class label’s value. Hence, a data balancing technique known as the Synthetic Minority Oversampling Technique (SMOTE) was used. SMOTE randomly generates new observations of the minority class to bring the number of minority class closer to the majority class [25,26]. A randomisation is also used during data balancing process to ensure the minority value of the class is randomly distributed throughout the dataset, and that the newly added observations of 1s do not gather in certain folds. The results of data balancing are presented in the final right column of Table 2 with a higher number of minority instances inserted to bring it closer to a 50–50 class ratio.

### 2.4. Feature Selection

Firstly, a feature-to-feature assessment was conducted without the class attribute, using the Pearson correlation matrix [27] to gain an understanding of the relationship between the cognitive items. This is to establish their dependency towards the class attribute, meaning a high correlation between two items will almost have the same effect on the dependent attribute, thus having the same properties towards the class. The items are simultaneously referenced against their DSM-5 cognitive domains to understand their sensitivity towards AD diagnosis. This will further assist our analysis when performing FS to derive a variety of subsets that covers a mixture of DSM-5 cognitive domains.

A range of FS techniques such as Information Gain (IG), Chi Squared Testing (CST) and ReliefF [28,29,30]; were utilised due to their different mathematical formulas to describe feature relevancy and have been successfully applied in previous dementia related studies such as [5,6,11,31]. The results obtained from the FS methods will each produce different scale of weightings, as such normalising the results is necessary to maintain a common scale and simplify the analysis. The assessment of features to keep or omit was based on different criteria including:

1. The ranking position of the features based on the relevancy score calculated by the FS methods
2. Identify clusters by observing for a distinct % drop pattern of the scores between subsequent features, from top to bottom ranking order per the below mathematical formula [5]
  $$ScoreDrop\in \%\left(Si,Si+1\right)=\frac{\left(Si-Si+1\right)}{Si}$$
3. Similarity of features is identified based on feature-to-feature assessment where a low intercorrelation is preferred.
4. Common features and their position ranking among the FS methods

The experiments evaluating the cognitive items will each result in five unique subsets as shown in Table 3. The formed subsets can provide clinicians an indication of the most sensitive features and its association with the DSM-5 cognitive domains that can assist in early screening of AD progression.

### 2.5. Classification

With the formed new features subsets, the next phase in the methodology is to evaluate these subsets using supervised learning algorithms to produce classification models. The classification stage consists of two phases, where the (1) first phase will only involve the derived subsets, while the (2) second phase will involve both the subsets and their demographic features (age, gender, level of education, race category, marital status) which is obtained from the ADNIMERGE dataset because of the data integration.

In the first phase, each subset will be evaluated using different classification algorithms including Bayes Net: Bayesian Network, LR: Logistic Regression, and C4.5: Decision Tree [32,33,34], where they each have unique training scheme to form the classification model and were successfully applied in previous dementia research such as [6,11,13]. A comparison analysis across the different classifiers will be conducted to evaluate the performance of the models based on different subsets, using measures such as the predictive accuracy, specificity, and sensitivity. In the second phase, the demographic features are included with the subsets and the same classification algorithms are again applied to assess the difference in model performance when demographics are included. Based on the comparison result analysis, the best performing model is selected.

## 3. Experimental Settings

Experimental were run utilising two open-sourced software which are the Waikato Environment for Knowledge Analysis (Weka) and The R Project for Statistical Computing (R), where both platforms have extensive data pre-processing, statistical and graphical tools, as well as machine learning algorithms for data analysis [35,36].

Using R version 1.3.1056, feature-to-feature correlation within the datasets is assessed to identify highly correlated items to derive subset number 2 of the ADNIMERGE-ADAS dataset. The ‘corrplot’ package ‘corr’ function plots the graph of the correlation matrix with coefficients to signal the strength between two items. The ‘caret’ package ‘findCorrelation’ function identified highly correlated items by calculating the largest mean absolute correlation between each item to remove any redundant features. Moreover, Pearson Correlation [27] is the default correlation method used within these functions. An implementation version of this method is employed as a function within Caret R package, which generates a correlation matrix of the data’s attributes as a vector of integers corresponding to columns in order to reduce pair-wise correlations [35]. When two features are correlated, the function checks at the mean absolute correlation of each feature and discards the feature with the biggest mean absolute correlation. A suggested Cut-off value of the correlation of 0.60 is used [37].

For the feature assessment, data balancing, and classification experiments, Weka 3.8.4 was used. When running experiments, 10-fold cross validation was employed to reduce the overfitting. 10-fold cross validation guarantees that the training dataset is partitioned into 10 stratified partitions randomly where the algorithm trains on 9 folds and then it geta evaluated on 1 repeatedly for 10 times in order to generate fair results. The implementation of the FS methods in WEKA were used and without amending the algorithms’ hyper parameters.

To measure the performance of the AD progression models derived by the classifiers against the subsets of cognitive features a number of standard evaluation metrics in machine learning including predictive accuracy, sensitivity, and the specificity [12,22] were utilised. Sensitivity is the measure of the proportion of actual positive cases that got predicted as positive. Specificity is the measure of how well a test can identify the true negatives, whilst accuracy is the measure of the correct classification of the instances based on models and measures.

All experiments have been conducted on a computing machine with Intel^®^ Core™ i5-6200U 2.3 Ghz with 8 GB RAM, on a Windows 10 Home, 64-bit. The hyperparameters of all feature selection methods and classification algorithms remained unchanged in the Weka platform.

## 4. Results

Experiments have been conducted using FS and classification methods against the ADNIMERGE-ADAS dataset, to evaluate and identify the most impactful cognitive items that can diagnose AD progression. The FS results analysis is based on criteria to derive potential effective subsets of cognitive features that may trigger the progression of AD, and their association with the DSM-5 diagnostic areas related to dementia. The first subset of features in each component will contain all test items which will serve as a baseline to allow for performance comparison against other subsets. The classification results will detail the predictive performance of the cognitive subsets and the process of selecting the best performing classification model.

### 4.1. Feature Selection Results

**Figure 2:**
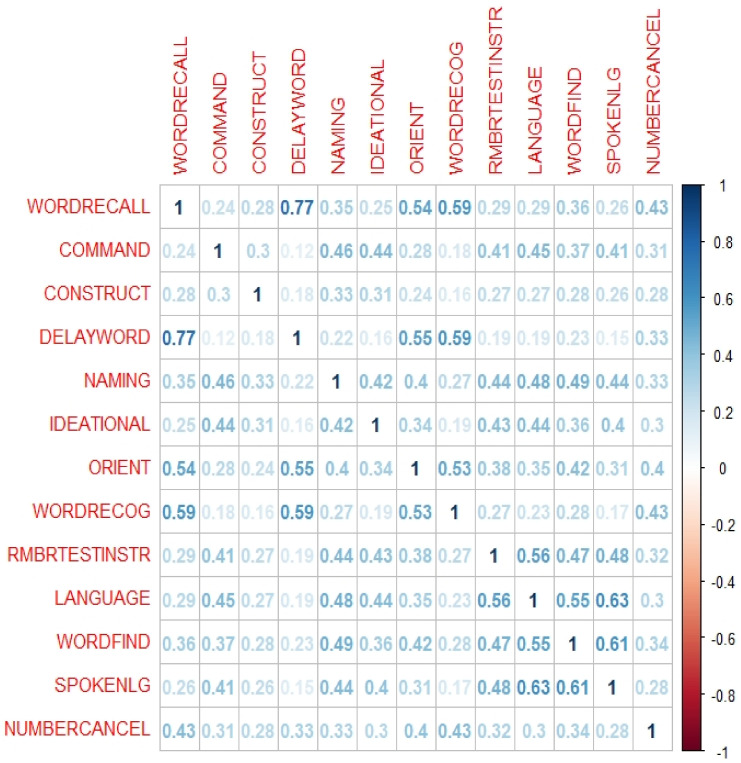
Pearson Correlation Coefficient Matrix of ADAS-Cog13 items.

From the ADNIMERGE-ADAS dataset, five unique subsets of cognitive items were derived (Table 4) using the various FS methods based on criteria described in Table 3. ADAS-subset2 was derived based on the Pearson Correlation coefficient matrix (Figure 2) with a cut-off of 0.6. ADAS-subset5 produced similar results as ADAS-subset3, it will not be required to be tested in the classification process. The computed scores of the FS results are normalised, then averaged as illustrated in Table 5, and then ranked in Table 6 from highest to lowest to measure the % drop in between the features. 

### 4.2. Classification Results

Using the derived subsets of distinct subsets of features from ADAS-Cog13 dataset, we evaluate their goodness by building predictive models using dissimilar classification algorithms (Bayes Net, LR, and C4.5). The difference in the number of features in each subset and their DSM-5 cognitive domain coverage will assist us when comparing the classification model performance measures such as predictive accuracy, sensitivity, and specificity. Table 7 (left side) illustrates the performance of the classification algorithms against each subset of ADAS-Cog13 excluding demographic features, only using the derived features and the class.

## 5. Results Discussion

### 5.1. Cognitive Feature Ranking

Based on feature-to-feature graph (Figure 2), cognitive items including word recall, word-finding, and language comprehension were found to have the highest correlation against other features where their coefficients are above the cut-off point. For example, word recall and word delay have a correlation of 0.77 against each other, signalling a strong correlation between both items, where they both have the same influence on the class attribute. As a result, one item between the two can be removed, and in this case, word recall has been identified as having a larger mean absolute correlation against the rest of the ADAS items and is thus removed. The properties of the word recall, word-finding, and language comprehension have the highest contributing factor to causing a high correlation when compared against each ADAS item. While these three features can be made redundant, the ten remaining features will still have the same effect on the class attribute. The removal of these items reduces overlapping of feature properties. The redundant items also have overlapping DSM-5 cognitive domains which are learning and memory, language, and complex attention, whereby the remaining items also cover these domains. However, ADAS-subset2 is only three features lesser than the original 13 items ADAS-Cog13, thus does not significantly reduce assessment and computational time.

Using the results of the FS methods of Table 5 and Table 6, three clusters were identified by observing a distinct drop pattern, in this case when there is a drop of >30% in the scores of two successive features. With the identified cluster groups, the features within cluster 1 were used as ADAS-subset3, which consists of word recall, delayed word recall and word recognition, covering two DSM-5 cognitive domain of learning and memory, and language. For ADAS-subset4, all features within clusters 1 and 2 were combined, bringing the subset to six items by adding orientation, command and word finding. ADAS-subset4 covers four out of six of the cognitive domains, which are learning and memory, language, executive function, and perceptual motor function. The remaining clusters are made redundant due to their lower effect on the class attribute.

The different FS methods used have identified three items that occurred the greatest number of times within each of the derived subsets. They are word recall, delayed word recall and word recognition, signalling its significant influence on predicting AD progression. These three items have the common association with retrieving words that require a patient to read, remember, recall, and recognise, and thus tap into the learning and memory, and language cognitive domains. During an assessment, a clinician can specifically pay attention to the performance of these tasks by the patient and determine whether there are signs of the disease progression and if the patient will need early intervention.

The results of the feature analysis showed that there is no cognitive feature that stood out, but we can identify sets of cognitive items that can be the assessed by clinicians while performing clinical assessment. For instance, correlations among cognitive items including word recall, word delay, word-finding, and language comprehension can be used by clinicians in cognitive assessment for dementia diagnosis.

In addition, the results reveal the three cognitive items (word recall, word recall delayed, and word recognition) are effective specially when processed by learning algorithms since models derived are within the medically accepted performance rates. These cognitive items cover two cognitive domains learning and memory, and language in the DSM-5 framework. When an additional cognitive feature is considered, orientation, the DSM-coverage increase to four domains: learning and memory, language, executive function, and perceptual motor function.

The AD advancement models derived by classification techniques such as LR, BayesNet, and Decision Tree can indeed cut down the clinical time during dementia diagnosis assessment since it is a lengthy—process where individuals under assessment may encounter fatigue. In addition, clinician can focus on certain cognitive assessments related to memory, orientation and communication which can help in forming specific intervention plans based on the level of cognitive impairment of the patients and the cognitive domain. More importantly, dementia progression models can also be integrated with digital platforms to enable fast access of information especially in the time of the pandemics where resources are overutilised thus reduction of assessment time is crucial.

### 5.2. Cognitive Feature Significance Using Classification Models

The results against the ADAS subsets identified during the feature assessment analysis and without using any demographic features showed superiority of C4.5 algorithm. To be exact, the AD progression models derived by C4.5 algorithm all have acceptable performance even when processing only three cognitive items (word recall, delayed word recall, word recognition) with 87.60% sensitivity rate. This rate is just 1.40% lesser than that when the same algorithm processed the complete ADAS items (ADAS-subset1). It seems that using only 3 cognitive items of ADAS that belong to the learning and memory and language DSM cognitive domains can indeed help clinicians in detecting AD progression at least using decision tree algorithms. These 3 items are also common items that appear in the other derived subsets, underlining its importance in predicting AD progression. The ability for the C4.5’s model to predict AD progression correctly is an important measurement due to the necessity of predicting a diagnosis correctly against true diagnosis, especially in the medical field.

While using the C4.5 algorithm, models derived from ADAS-subset2 produced the best accuracy and specificity measures; and models derived from the ADAS-subset4 produced the best sensitivity measure, the difference in percentages across all measures against ADAS-subset 3 is not high. The fact that when only 3 cognitive items are processed can produce good results meaning less assessment and computation time is required. Furthermore, models derived from ADAS-subset3 achieved this despite that this subset of data only cover only two DSM-5 prescribed cognitive domains, in comparison to ADAS-subset2 and ADAS-subset4 that cover five and four cognitive domains, respectively. Interestingly, the overall performance against the ADAS data subsets produced similar results conducted by other ADAS validation studies where the accuracy ranged from 82–99.6%, sensitivity from 58–74% and specificity from 91–98% [22,23].

When demographic features (age, gender, level of education, race category, marital status) are included in the data analysis (right side of Table 7) with each ADAS items, the performance across all classification algorithms improved, particularly evident in the Bayes Net and LR algorithms with at least >10% improvement across all performance measures. While the C4.5 algorithm also improved though not as significantly, it was still superior in comparison to the other two algorithms overall as it further elevated when processed ADAS-subset4’s (WORDRECALL, DELAYWORD, WORDRECOG, ORIENT, COMMAND, WORDFIND) reaching a performance to above 90%, whereby it was able to achieve an accuracy of 91.94%, sensitivity of 92.60% and specificity of 91.20%. The model derived by the C4.5 algorithm from ADAS-subset4 can achieve better performance with just 6 ADAS items covering four out of six of the DSM-5 cognitive domains which are learning and memory, language, executive function, and perceptual motor function.

Overall, models produced from ADAS-subset4 with additional demographics features by the machine learning algorithms generate good performance and save time with only requiring assessment in 6 out of the 13 ADAS items. While the C4.5 algorithm may not have produced the best results across all measures, its overall results are above 90%, falling within the performance range of other ADAS-cog validation studies [22,23] and required the use of lesser features, as a result, it can be classified as a good performing model.

Since Decision Tree algorithms like C4.5 provide classification models with rules then such models can be utilised by clinicians during the dementia diagnosis process. These models are easy to be understood by the clinicians since they show relationships among cognitive items. In addition, clinicians can use a digital information toolkit that contains these cognitive items, their mapping to the DSM-5 neurocognitive domains to guide them in any diagnostic-related decisions or the preparation of intervention plans. More importantly, the output models of the C4.5 algorithm can also detect the disease progression for individuals undertaking a clinical assessment.

Offering models with rules by automated techniques like C4.5 empowers the clinicians to not only better understand each case’s context during the clinical evaluation but also comply with the in the General Data Protection Regulation (GDPR) regarding decision-making [5]. The outcome presented as a digital information toolkit provides the clinicians the ability to answer the questions related to the diagnostic process asked by the patients and family members on cognitive features that have contributed to the dementia conditions or the disease advancement, if any. These models aligns with the patient’s ‘right for an explanation’ as outlined in the GDPR since the process of deriving them is automated based on computational intelligence or machine learning techniques.

Analysing the classification models in regard to the influence of demographics features showed that the cognitive items mainly covering the cognitive domains of learning and memory, and language, when processed by classification techniques, produced models that performed well with the inclusion or exclusion of demographic features. When investing further specific demographic features like age, education level and gender, age seems associated with AD as the critical factor followed by gender and education level. When age and education level are included with cognitive items, the classification models generated by the algorithms have improved regarding evaluation metrics considered. The descriptive analytics results pinpoint that cognitive impairment often unfold in the early stages of the disease. Moreover, people with higher educational levels tend to be at higher risk of the disease progression at least when using the datasets considered. However, these claims require further in depth investigation using predictive analytics and using specific cohorts like individuals with baseline CN who progressed to MCI within a particular timeline.

## 6. Conclusions

With an ageing population, the increase burden of economic cost on the dementia community, coupled with an ongoing pandemic, it is becoming more critical to develop a faster, cheaper, and reliable way of diagnosing AD progression. Current systems involving both pathological and neuropsychological assessments are often physical invasive, psychologically stressful, time consuming and costly. Furthermore, the availability of these assessments to lower-income countries and rural areas are even less accessible. Hence, our research into design and implement a data driven approach based on machine learning techniques that can predict AD progression in a manner that is accessible, easy, affordable, quick to perform, and does not require special and expensive resources. This research aims to determine the cognitive items that can trigger AD progression, using the ADNI data observations of the ADAS-Cog13 assessment, which are common methods for a clinician to perform for AD and MCI screening and diagnosis. The approach developed involve data understanding, mapping cognitive features to DSM-5, data integration, new data modelling, and experimental data analysis that incorporate various FS methods (Pearson correlation, IG, CST and ReliefF) and classification techniques (BayesNet, LR, C4.5).

In evaluating the cognitive items from the ADAS-Cog test using FS and classification techniques, the results revealed that word recall, delayed word recall and word recognition are influential items for AD progression as they have been identified by the FS methods and clustering analysis. These cognitive features tap into the learning and memory, and language domains; while these 3 items (ADAS-subset3) only cover two DSM-5 domains, C4.5 algorithm can produce relatively good model performance when processing them across the three evaluation measures at 82.90% accuracy, 87.60% sensitivity and 78.20% specificity, and with sensitivity being only 1.40% less than when the same algorithm processes the complete items of ADAS-Cog13 (ADAS-subset1). In fact, across all the ADAS subsets, the C4.5 algorithm provided relatively good performance with or without including demographics, while the BayesNet and LR algorithms only generated better results when including demographics to the cognitive features. When including demographics features the classification techniques were able to generate models with better results overall. To be exact, the AD progression models produced by the C4.5 model against ADAS-subset3 (3 items) when including demographics achieved 88.69% accuracy, 88.80% sensitivity and 88.60% specificity improving the model’s performance by 5.79% accuracy, 1.20% sensitivity and 5.70% specificity when compared to the model derived by the same algorithm from the same subset but without demographics.

In addition, the classification techniques showed competitive results when processing ADAS-subset 4 (word recall, delayed word recall, word recognition, orient, command, and word finding) that taps into four DSM-5 cognitive domains which are learning and memory, language, executive function, and perceptual motor function. In particular, C4.5 algorithm produced models with a superior sensitivity of 88.80% and when demographics are included with ADAS-subset 4 the sensitivity of the models produced by C4.5 algorithm improved by 3.80%. The same pattern is also observed with the other evaluation measures including accuracy and specificity at least for the cognitive subsets of ADAS.

In conclusion, while there was no single cognitive item that stood out individually, our research was able to identify sets of key cognitive items within the ADAS-cog13 assessment. The ADAS items mainly covering cognitive domains of learning & memory, and language, when processed by machine learning techniques, produce models that performed well with or without demographic features and showed superior models using C4.5 decision tree algorithm. Overall, cognitive items appear to be more influential that recently reported results on functional items of FAQ seeing that without demographic features, the decision tree algorithm model was still able to generate results across the dissimilar evaluation metrics that are above 80%.

Being able to identify the items that have the highest impact on the progression of the AD will assist the clinician not only in time and cost-savings, but also provide the chance of early detection, a shorten and quick-to-deploy information sheet on AD diagnosis, a better understanding of how cognitive items, can trigger any progression of AD.

A limitation towards our study is that it does not take into consideration the time taken for the disease to progress from CN to MCI or MCI to AD. In near future, we will perform a longitudinal characteristic of the dataset to present new insights into how long it takes for the disease to progress. We also want to determine which stage of the disease the patient is going through and the different types of treatment that can be given.

Another limitation of this study is that it did not distinguish between individuals with progression from CN to MCI and other cohorts like individuals’ with progression from MCI stage to AD. Thus, in near future, we intend to isolate patients and controls that fall within the above two progression categories and study their progression within a specified timeframe.

## Figures and Tables

**Table 1:** General statistics of the datasets before pre-processing.

Name of the Dataset	# of Items	# of Participants	# Data Examples	Any Missing Values
ADNIMERGE	113	2260	14,627	Diagnostic Class (DX): 4243
ADAS-Cog	121	1751	6770	100 missing values: 91 across ADAS tasks and 9 in VISCODE2 attribute

**Table 2:** Data statistics.

Dataset Name	# of Features	# of Patients	# of visits (Data Examples)	DX Statistics Before Balancing the Dataset	DX Statistics after Balancing the Dataset
ADNIMERGE-ADAS	24	1710	6330	Total observations: 6330 ‘0’: 6020 (majority 95%) ‘1’: 310 (5%)	Total observations: 11,943 ‘0’: 6020 (50.4%) ‘1’: 5923 (49.6%)

**Table 3:** Summary of the methods used to derive each subset.

Subset	Derived Method
**1**	All Items in each screening method
**2**	Pearson correlation method
**3**	Cluster #1—derived from a composite normalised average weighting of three FS filter methods: IG, CST and ReliefF.
**4**	Cluster #1 + #2—derived from a composite normalised average weighting of three FS filter methods: IG, CST and ReliefF.
**5**	Common top three features identified by the FS methods

**Table 4:** Summary of the derived ADAS-Cog13 items for each subset.

Subset	Derived ADAS-Cog13 Items	FS Criteria
ADAS-subset1	All ADAS-Cog13 items (13 items)	-
ADAS-subset2	COMMAND, CONSTRUCT, DELAYWORD, NAMING, IDEATIONAL, ORIENT, WORDRECOG, RMBRTESTINSTR, SPOKENLG, NUMBERCANCEL	Remove highly correlated items based on the Pearson correlation matrix
ADAS-subset3	WORDRECALL, DELAYWORD, WORDRECOG	Cluster analysis based on % drop pattern
ADAS-subset4	WORDRECALL, DELAYWORD, WORDRECOG, ORIENT, COMMAND, WORDFIND	Cluster analysis based on % drop pattern
ADAS-subset5	WORDRECALL, DELAYWORD, WORDRECOG	Common features in the FS methods’ results

**Table 5:** ADAS-Cog13 items with computed scores and normalized scores derived by the FS methods from (ADAS-subset3 & ADAS-subset4).

Feature	IG		CST		ReliefF		Average Scores
	Score	Normalised	Score	Normalised	Score	Normalised	
WORDRECALL	0.135	1.000	1956.268	1.000	0.144	0.683	0.894
COMMAND	0.049	0.246	699.447	0.242	0.026	0.100	0.196
CONSTRUCT	0.032	0.093	475.613	0.107	0.030	0.121	0.107
DELAYWORD	0.084	0.551	1327.623	0.621	0.208	1.000	0.724
NAMING	0.032	0.101	472.299	0.105	0.022	0.080	0.095
IDEATIONAL	0.038	0.150	534.736	0.143	0.015	0.047	0.113
ORIENT	0.044	0.201	691.697	0.237	0.081	0.372	0.270
WORDRECOG	0.062	0.364	975.468	0.408	0.172	0.826	0.533
RMBRTESTINSTR	0.021	0.002	298.131	0.000	0.005	0.000	0.001
LANGUAGE	0.028	0.059	389.760	0.055	0.009	0.016	0.043
WORDFIND	0.042	0.182	629.659	0.200	0.035	0.147	0.176
SPOKENLG	0.025	0.033	347.811	0.030	0.009	0.020	0.028
NUMBERCANCEL	0.021	0.000	314.056	0.010	0.060	0.268	0.092

**Table 6:** Cluster items identified based on the % drop pattern between the ranked items as according to their normalised average scores (ADAS-subset3 & ADAS-subset4).

Feature by Rank	Normalised Average Scores	% Drop after Normalisation	Cluster No.
WORDRECALL	0.894		Cluster #1
DELAYWORD	0.724	19.07%	Cluster #1
WORDRECOG	0.533	26.41%	Cluster #1
ORIENT	0.270	49.29%	Cluster #2
COMMAND	0.196	27.47%	Cluster #2
WORDFIND	0.176	10.17%	Cluster #2
IDEATIONAL	0.113	35.66%	Cluster #3
CONSTRUCT	0.107	5.45%	Cluster #3
NAMING	0.095	10.90%	Cluster #3
NUMBERCANCEL	0.092	3.13%	Cluster #3
LANGUAGE	0.043	53.22%	Cluster #4
SPOKENLG	0.028	36.03%	Cluster #5
RMBRTESTINSTR	0.001	97.89%	Cluster #6

**Table 7:** The classification algorithms performance against subsets of the ADAS-Cog13 items.

Subset	Algorithm	Without Using Any Demographics			When Using Few Demographics		
		Accuracy %	Sensitivity %	Specificity %	Accuracy %	Sensitivity %	Specificity %
**ADAS-subset1 (baseline)**	**BayesNet**	79.27%	84.30%	74.30%	88.51%	89.60%	87.50%
	**Logistic Regression**	81.83%	86.10%	77.70%	91.01%	93.60%	88.50%
	**C4.5**	88.59%	89.00%	88.20%	91.48%	91.60%	91.40%
**ADAS-subset2**	**BayesNet**	73.81%	77.50%	70.20%	87.87%	90.40%	85.30%
	**Logistic Regression**	77.07%	81.90%	72.40%	90.26%	93.30%	87.30%
	**C4.5**	86.68%	86.90%	86.40%	91.75%	91.50%	92.00%
**ADAS-subset3**	**BayesNet**	69.80%	78.10%	61.60%	78.80%	84.90%	72.80%
	**Logistic Regression**	74.21%	78.80%	69.70%	80.05%	83.50%	76.60%
	**C4.5**	82.90%	87.60%	78.20%	88.69%	88.80%	88.60%
**ADAS-subset4**	**BayesNet**	74.36%	78.20%	70.50%	87.37%	89.10%	85.60%
	**Logistic Regression**	79.40%	84.10%	74.80%	90.35%	93.10%	87.60%
	**C4.5**	86.19%	88.80%	83.60%	91.94%	92.60%	91.20%

## Data Availability

Data are available at: https://adni.loni.usc.edu/.
